# Expression of CK17 and SOX2 in Vulvar Intraepithelial Neoplasia: A Comprehensive Analysis of 150 Vulvar Lesions

**DOI:** 10.3390/cancers16233966

**Published:** 2024-11-26

**Authors:** Nikki B. Thuijs, Féline O. Voss, Patricia C. Ewing-Graham, Shatavisha Dasgupta, Johannes Berkhof, Johan Bulten, Koen van de Vijver, Maaike C. G. Bleeker

**Affiliations:** 1 Department of Pathology, Amsterdam UMC Location Vrije Universiteit Amsterdam, De Boelelaan 1117, 1081 HV Amsterdam, The Netherlands; n.thuijs@amsterdamumc.nl (N.B.T.); f.o.voss@amsterdamumc.nl (F.O.V.); 2 Cancer Center Amsterdam, Imaging and Biomarkers, 1081 HV Amsterdam, The Netherlands; 3 Department of Pathology, Erasmus MC, Dr. Molewaterplein 40, 1016 LV Rotterdam, The Netherlands; p.ewing@erasmusmc.nl; 4 Broad Institute of MIT and Harvard, Imaging Platform, 415 Main Street, Cambridge, MA 02142, USA; sdasgupt@broadinstitute.org; 5 Epidemiology and Data Science, Amsterdam UMC Location Vrije Universiteit Amsterdam, De Boelelaan 1117, 1081 HV Amsterdam, The Netherlands; h.berkhof@amsterdamumc.nl; 6 Department of Pathology, Radboud University Medical Center, Geert Grooteplein Zuid 10, 3769 AL Nijmegen, The Netherlands; hans.bulten@radboudumc.nl; 7 Department of Pathology, Cancer Research Institute Ghent, Ghent University Hospital, Corneel Heymanslaan 10, 9000 Ghent, Belgium; koen.vandevijver@uzgent.be

**Keywords:** diagnostic accuracy, CK17, SOX2, HPV-independent VIN, vulvar HSIL

## Abstract

Background: Recently, the immunohistochemical markers cytokeratin 17 (CK17) and SRY-box2 (SOX2) have been evaluated as adjuncts for the diagnosis of high-grade vulvar intraepithelial neoplasia (VIN). In the present study, the aim was to assess CK17 and SOX2 expression in VIN by studying 150 vulvar lesions, originally reported as high-grade VIN and to assess the diagnostic accuracy. Methods: All slides (H&E, p16^INK4a^, p53, Ki-67, CK17, and SOX2 stains) were independently assessed by six pathologists and the final diagnosis was reached in consensus meetings, as follows: 46 human papillomavirus (HPV)-independent VIN (including 30 p53 mutant and 16 p53 wild-type lesions), 58 high-grade squamous intraepithelial lesions (HSILs), 4 low-grade SILs (LSILs), 37 non-dysplastic lesions, and 5 lesions where the histology was inconclusive. Results: CK17 positivity was observed in 100% p53 wild-type HPV-independent VIN, compared to 73% p53 mutant HPV-independent VIN, 14% HSILs, 0% LSILs, and 24% non-dysplastic lesions. SOX2 positivity was observed in 13% p53 wild-type HPV-independent VIN, 43% p53 mutant HPV-independent VIN, 2% HSILs, 0% LSILs, and 3% non-dysplastic lesions. The highest diagnostic accuracy (89%) for HPV-independent VIN was obtained when combining p53 and CK17 immunohistochemistry. The addition of SOX2 did not further increase diagnostic accuracy. Conclusion: To conclude, aside from p53, both CK17 and SOX2 can be of value for reaching an accurate diagnosis of HPV-independent VIN.

## Simple Summary

High-grade vulvar intraepithelial neoplasia (VIN) is subdivided into human papillomavirus (HPV)-associated high-grade squamous intraepithelial lesions (HSILs) and HPV-independent VIN. The diagnosis of HPV-independent VIN can be very challenging. In this study, the potential diagnostic value of cytokeratin 17 (CK17) and SRY-box2 (SOX2) immunohistochemistry was assessed in a series of 150 vulvar lesions, originally diagnosed as high-grade VIN and re-assessed by six pathologists (including H&E, p16^INK4a^, p53, Ki-67, CK17, and SOX2 immunohistochemistry). The diagnostic accuracy of the markers was calculated for the diagnosis of HPV-independent VIN, using non-dysplastic cases as controls. Significantly more CK17- and SOX2-positive cases were observed in HPV-independent VIN compared to non-dysplastic cases (*p* < 0.001). The highest diagnostic accuracy (89%) for HPV-independent VIN was obtained when combining the p53 and CK17 immunohistochemical markers. In conclusion, this study confirms that, in addition to p53, both CK17 and SOX2 can have added value in the diagnostic work-up of lesions suspected of HPV-independent VIN.

## 1. Introduction

High-grade vulvar intraepithelial neoplasia (VIN) is subdivided into human papillomavirus (HPV)-associated high-grade squamous intraepithelial lesions (HSILs) and HPV-independent VIN, with the latter usually clinically referred to as differentiated VIN (dVIN) based on the WHO 2020 classification of female genital tumors [1]. Recent studies proposed the further subdivision of HPV-independent VIN into the following two prognostically significant groups: p53 mutant and p53 wild-type lesions [2,3]. Histopathological variants of p53 wild-type HPV-independent VIN include vulvar acanthosis with altered differentiation (VAAD), differentiated exophytic vulvar intraepithelial lesion (DEVIL), and verruciform lichen simplex chronicus, which have all recently been described and grouped under the term verruciform acanthotic VIN (vaVIN) [4]. The International Society of the Study of Vulvovaginal Diseases (ISSVD) recognizes the term vulvar aberrant maturation (VAM) to include DEVIL, VAAD, and other related p53 wild-type lesions [5]. Given the low incidence of HPV-independent VIN, the poor reproducibility and overlapping morphology, the terms VAAD, DEVIL, vaVIN, and VAM are not consistently applied. More importantly, this morphological subtyping does not reflect the biological nature in terms of cancer risk, and thus the usefulness of these terms in clinical practice can be questioned.

An accurate diagnosis of high-grade VIN and the distinction between HSILs and HPV-independent VIN are important, considering the implications for treatment and prognosis [2,6]. The 10-year risk of progression to invasive cancer is 67% for p53 mutant HPV-independent VIN, whereas this is 28 to 37% for p53 wild-type HPV-independent VIN, DEVIL, VAAD, and verruciform lichen simplex chronicus, and 6% in HSILs [2,7]. However, the diagnosis of HPV-independent VIN with a p53 wild-type staining pattern is particularly challenging [8]. Owing to limited cytonuclear atypia and overlapping histomorphological features, p53 wild-type HPV-independent VIN may be misdiagnosed as a non-dysplastic or reactive lesion, or as a low-grade squamous intraepithelial lesion (LSIL). Hence, there is a need for additional diagnostic markers to help differentiate p53 mutant HPV-independent VIN from its mimics.

Few studies have shown the potential value of the immunohistochemical markers CK17 and SOX2 in VIN [9,10,11,12,13,14]. Dasgupta et al. observed CK17- and SOX2-positive immunohistochemical staining in 81% and 86% of HPV-independent VIN and in 63% and 88% of HSILs, respectively, as compared to 9% and 20% of non-dysplastic lesions [10].

Cytokeratin 17 (CK17) is an intermediate filament protein that is induced in activated keratinocytes [15,16]. Previous investigations have demonstrated elevated CK17 expression in premalignant and malignant tissues compared to healthy tissues [17]. Sex-determining region Y-box 2 (SOX2) is located on chromosomal segment 3q26.33, serves as a critical regulator of pluripotent stem cells, and helps to maintain and develop the squamous epithelium [18]. Previous studies have indicated that SOX2 functions as an oncogene and is subject to highly recurrent genomic amplification in squamous cell carcinomas, including those of the anogenital region, lung, head and neck, and oral cavity [19,20].

The current study aimed to assess the expression rates of CK17 and SOX2 in VIN, as well as the contribution to diagnostic accuracy for the diagnosis of HPV-independent VIN.

## 2. Materials and Methods

### 2.1. Study Population and Categorization of Vulvar Lesions

From a population-based historical cohort consisting of 751 patients, all originally diagnosed with high-grade VIN, a subset of 150 cases were selected for the current study [2,21]. All 751 cases had previously been reviewed and, for the current study, all the cases of HPV-independent VIN, non-dysplastic lesions, and cases with inconclusive histology were selected, along with all the HPV-associated cases that were present on the slides used for immunohistochemistry of the aforementioned cases [2].

All 150 patients in the current cohort had no vulvar cancer history and no concurrent vulvar cancer at the time of high-grade VIN diagnosis. The HPV DNA test result was available for each case. Follow-up information on the progression to vulvar cancer was available from the nationwide registry of histopathology in the Netherlands [22]. Selected cases included part of the HPV-associated lesions and all HPV-independent lesions (i.e., HPV-independent VIN and non-dysplastic lesions), as well as all inconclusive cases, as concluded after a previous histopathological revision [2].

Using a data collection form and the PathXL online viewer, H&E and immunohistochemical slides were scored independently by five pathologists and one resident in pathology (N.B.T., K.V.D.V., P.C.E.G., S.D., J.B., and M.C.G.B), all with a high exposure to vulvar pathology. The final diagnosis was based on H&E, p16^INK4a^, p53, Ki-67, and HPV results. Vulvar lesions were categorized as HPV-associated (HSIL or LSIL), HPV-independent VIN, or non-dysplastic lesions, such as lichen sclerosus (LS), reactive lesions, and other non-dysplastic dermatoses. Discrepancies in the final diagnosis or immunohistochemical staining patterns were discussed in consensus meetings.

### 2.2. Tissue Processing

Details of the tissue processing, immunohistochemistry of p16^INK4a^, p53, and Ki-67, DNA isolation, and HPV DNA testing have been described previously [2]. Immunostaining for CK17 and SOX2 was performed with the Optiview detection kit, with the automated 100 BenchMark ULTRA IHC/ISH system (Roche) and with mouse monoclonal antibodies against the keratin17 antigen (clone SP95; Abcam, Cambridge, UK) and the SOX2 antigen (clone EP103; Cellmarque, Rocklin, CA, USA).

### 2.3. Immunohistochemical Staining Patterns for p16^INK4a^, p53, Ki-67, CK1, and SOX2

**Figure 1:**
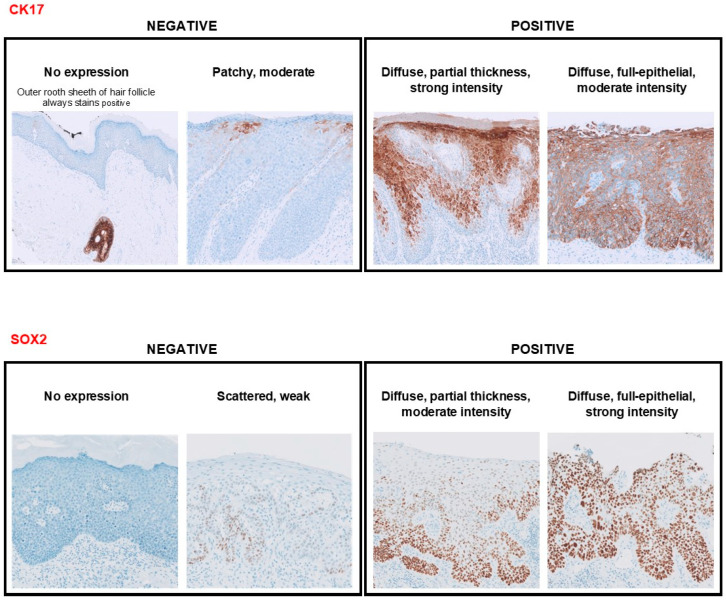
Representative examples of CK17 and SOX2 immunohistochemical staining patterns. CK17 and SOX2 staining patterns were categorized as negative (no expression, patchy or scattered staining, or staining with weak intensity) or positive (diffuse and moderate-to-strong intensity). CK17 is positive in the outer root sheath of the hair follicle epithelium.

P16^INK4a^ staining was scored as negative (absent or patchy) or block (diffuse) positive (≤1/3, ≤2/3, >2/3). P53 staining was scored as wild type (scattered or mid-epithelial with basal sparing) or mutant (nuclear positive, null or cytoplasmic positive). Ki-67 staining was scored as not increased (a few positive parabasal nuclei) or increased (≤1/3, ≤2/3, >2/3) [2]. CK17 cytoplasmic staining was scored in the horizontal direction as patchy (<50%) or diffuse (>50%), and SOX2 nuclear staining as scattered (<50%) or diffuse (>50%) [10]. For both CK17 and SOX2, staining in the vertical direction was scored as either full-epithelial or partial thickness. Staining intensity was recorded as mild, moderate, or strong. Subsequently, for purposes of statistical analysis, the features of CK17 and SOX2 staining described above were combined, resulting in the following two final categories, similar to those used previously by Dasgupta et al: negative (no expression, patchy or scattered staining, or staining with weak intensity) or positive (diffuse and moderate-to-strong intensity) (Figure 1) [10].

### 2.4. Statistical Analysis

Immunohistochemistry scores for each disease category were compared using Pearson’s Chi-Squared test. The level of statistical significance was set at 0.05. The performance of p53, CK17, and SOX2 immunohistochemical markers was calculated for the diagnosis of HPV-independent VIN using non-dysplastic lesions as a control group. This included sensitivity, specificity, and accuracy calculations. These calculations included a 95% confidence interval (95% CI). Statistical analysis was performed using IBM SPSS Statistics software for Windows version 28.0 (IBM Corporation, Armonk, NY, USA).

## 3. Results

### 3.1. Vulvar Disease Categories

After revision of the H&E images and immunohistochemistry by the participating pathologists, consensus was reached on the diagnoses for the study cohort, which comprised 46 HPV-independent VIN (30 p53 mutant and 16 p53 wild-type), 58 HSILs, 4 LSILs, 37 non-dysplastic lesions, and 5 inconclusive lesions. Non-dysplastic lesions included LS (n = 7), inflammation (n = 11), reactive changes (n = 12), (fibro-)epithelial polyps (n = 2), and vulvar tissue without histological abnormalities (n = 5).

The immunohistochemical expression of the markers in relation to the final diagnoses are shown in Table 1.

Block-positive p16^INK4a^ was observed in all the HSILs (100%) and in 1/46 (3%) of the HPV-independent VIN (*p* < 0.001). The single p16^INK4a^ block-positive HPV-independent VIN demonstrated p53 mutant staining and tested negative for HPV. Mutant p53 staining was observed in 30/46 (65.2%) of the HPV-independent VIN. One lesion in this series did not meet the criteria for HPV-independent VIN based on histomorphology but did demonstrate a p53 mutant staining pattern. This case was categorized as inconclusive. None of the 37 non-dysplastic lesions displayed mutant p53 staining.

### 3.2. CK17 Immunohistochemistry

CK17 expression patterns differed significantly across all disease categories (*p <* 0.001) (Table 1). Positive CK17 staining (i.e., a diffuse, moderate-to-strong staining pattern) was seen in 38/46 (83%) of the HPV-independent VIN, including 22/30 (73%) of the p53 mutant HPV-independent VIN and all 16 (100%) of the p53 wild-type HPV-independent VIN. In the HSIL, LSIL, and the non-dysplastic lesions, lower CK17 positivity rates of 14%, 0%, and 24% were observed, respectively.

**Figure 2:**
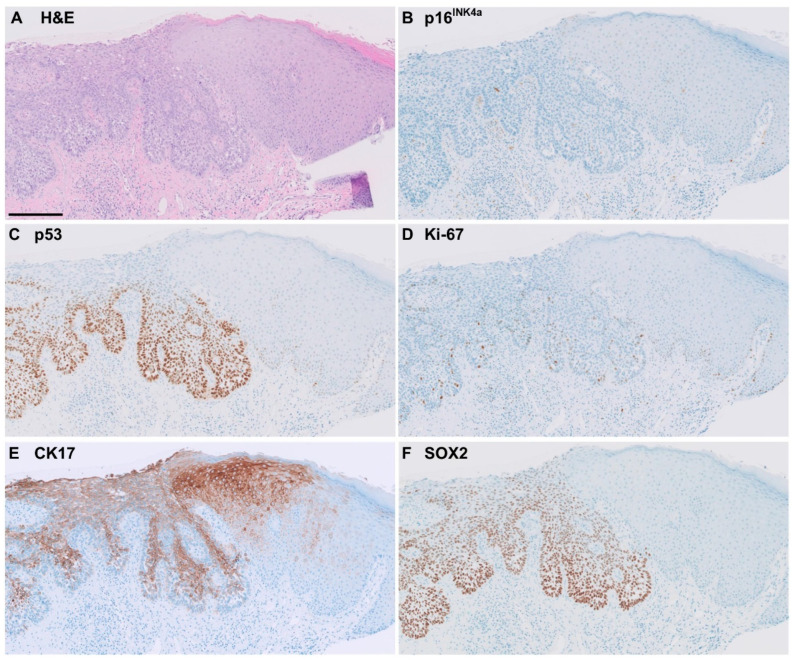
Example of immunohistochemical staining pattern observed in p53-mutant HPV-independent VIN, showing classical ‘differentiated’ features. Bar = 200 µm. (**A**) H&E staining. (**B**) Negative p16^INK4a^. (**C**) Positive p53 staining-mutant pattern. (**D**) No increased Ki-67. (**E**) Positive CK17, diffuse, moderate intensity and near full-epithelial expression. (**F**) Positive SOX2, diffuse, moderate-to-strong intensity, full-epithelial expression.

**Figure 3:**
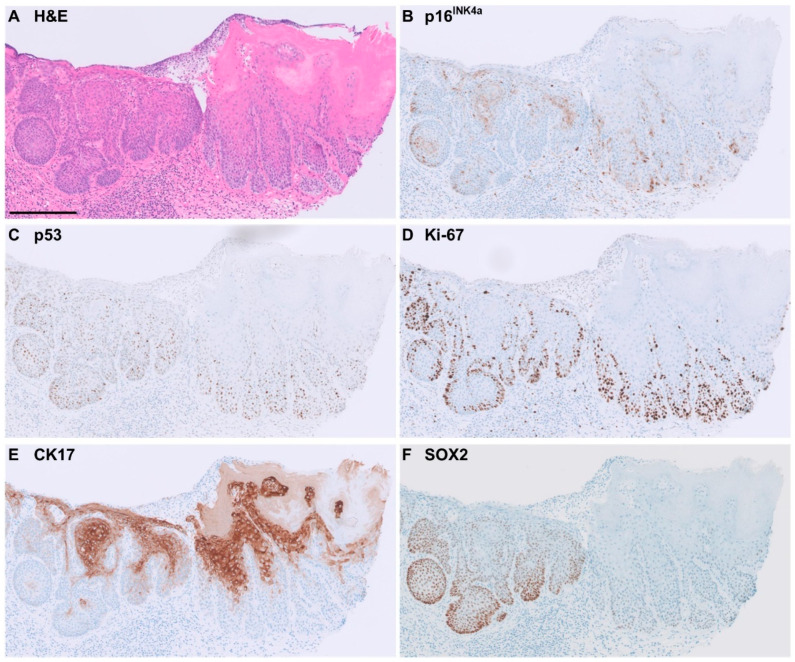
Example of immunohistochemical staining pattern observed in p53 wild-type HPV-independent VIN. Bar = 200 µm. (**A**) H&E staining. (**B**) Negative (patchy) p16^INK4a^. (**C**) Wild-type pattern of p53 staining. (**D**) Increased Ki-67 expression up to 1/3 of epithelial thickness. (**E**) Positive CK17, diffuse, moderate-strong intensity and partial thickness expression. (**F**) Positive SOX2, diffuse, moderate-strong intensity, partial thickness expression.

Of the 38 CK17-positive cases of HPV-independent VIN, 63% showed full-epithelial CK17 expression. CK17 staining was often seen in the more superficial keratinocytes showing differentiation. Representative examples of immunohistochemical staining patterns in HPV-independent VIN are shown in Figure 2 and Figure 3.

The CK17-positive non-dysplastic lesions (n = 9) included three cases of LS, two reactive lesions, two cases of hyperplasia, and two epithelial polyps. The CK17-negative non-dysplastic lesions (n = 27) included 11 lesions showing inflammation, 4 cases of LS, 10 reactive lesions, and all 5 normal epithelia. The p53 mutant inconclusive case showed an absence of CK17 staining.

### 3.3. SOX2 Immunohistochemistry

SOX2 expression patterns also differed significantly across all disease categories (*p <* 0.001) (Table 1). Positive SOX2 staining was seen in 15/46 (33%) of the HPV-independent VIN; in 13/30 (46%) of the p53 mutant HPV-independent VIN and in 2/16 (13%) of the p53 wild-type HPV-independent VIN (*p* = 0.023). The majority of HSIL, LSIL and non-dysplastic cases showed negative staining for SOX2. Only 2% of the HSILs showed positive staining, while none of the LSIL cases did so. In addition, only one non-dysplastic (reactive) lesion stained positive for SOX2. SOX2 was negative in the five vulvar epithelia without abnormalities.

Of all the SOX2-positive HPV-independent VIN, eight cases (53%) showed SOX2 expression across the full-epithelial thickness (Figure 2), and seven cases (47%) showed partial thickness staining (Figure 3).

### 3.4. Performance of Markers for Accurate Diagnosis of HPV-Independent VIN

Significantly more CK17 and SOX2 positivity was observed in HPV-independent VIN compared to non-dysplastic cases (*p* < 0.001). The performance of p53, CK17, and SOX2 in relation to the diagnosis of HPV-independent VIN is shown in Table 2. P53 and CK17 had a high accuracy for the detection of HPV-independent VIN (80% and 79%, respectively). Although SOX2 showed a high specificity of 97%, the low sensitivity of 33% resulted in a moderate accuracy for the detection of HPV-independent VIN of 61%.

Combining the markers p53 and CK17—in other words, where cases had to show either mutant p53 staining and/or positive CK17 staining—resulted in the highest accuracy for the diagnosis of HPV-independent VIN of 89%. Adding SOX2 to this combination did not further increase accuracy.

### 3.5. Prognostic Value of CK17 and SOX2

Of the 36 non-dysplastic lesions, 13 (35%) progressed to VIN during follow-up. Of these, five (42%) were positive with CK17 and one (8%) was SOX2 positive. Of the 24 non-dysplastic cases that did not progress to VIN, 4 (17%) were CK17 positive and none were SOX2 positive. Hence, a significant correlation between CK17 and SOX2 staining and progression to dysplasia could not be demonstrated (*p* = 0.102 and *p* = 0.151, respectively).

Within the total follow-up period of 23.3 years, 25/46 (54%) HPV-independent VIN progressed to vulvar cancer. Of the 14 cases that progressed to vulvar cancer within two years, 12 (86%) and 7 (50%) were found to be CK17 and SOX2 positive, respectively. Of the 32 cases that did not progress to cancer within two years, 26 (81%) and 8 (25%) were CK17 and SOX2 positive, respectively. A significant correlation between CK17 and SOX2 staining and progression to vulvar cancer within two years could not be demonstrated (*p* = 0.713 and *p* = 0.096, respectively).

## 4. Discussion

Our study demonstrated positive CK17 staining (i.e., a diffuse, moderate-to-strong staining pattern) in the majority of HPV-independent VIN (83%), with 73% in p53 mutant HPV-independent VIN and 100% in p53 wild-type HPV-independent VIN. This is consistent with the findings of others [11,12,14]. CK17 expression in VIN was first described in 2017, showing increased expression in up to 93% of HPV-independent VIN [14]. More recently, other research groups have reported an increased CK17 expression in HPV-independent VIN (81 to 100%), which is similar to our study. SOX2 demonstrated significantly more positive staining in p53 mutant HPV-independent VIN than in p53 wild-type HPV-independent VIN but showed lower accuracy for the diagnosis of HPV-independent VIN than CK17, owing to low sensitivity. Combining p53 and CK17 showed the highest accuracy for the diagnosis of HPV-independent VIN. The addition of SOX2 did not further increase the accuracy.

There is a clinical need for additional diagnostic markers for the accurate diagnosis of p53 wild-type HPV-independent VIN, given that the prognosis differs substantially from its mimickers [2]. Several studies have reported on CK17 expression in relation to p53 staining patterns in HPV-independent VIN [11,12,13,14]. Such stratified data were also provided to us by Dasgupta et al. [10]. Interestingly, when combining all p53 wild-type HPV-independent VIN cases from the four aforementioned studies, 57/62 (92%) of the cases showed positive CK17 expression, comparable to the 100% positivity rate observed in this series. When combining all p53 mutant HPV-independent VIN cases from these studies, 72/81 (89%) of the cases demonstrated positive CK17 expression, which is a little higher than the 73% found in our study. One possible explanation for this difference is the varying proportion of cases with concurrent vulvar cancer between these studies.

CK17 exhibits a diffuse and moderate-to-strong expression in most cases of p53 wild-type HPV-independent VIN. In particular, when combined with p53, the specificity of CK17 is high, i.e., if CK17 is negative in a p53 wild-type lesion, this argues against an HPV-independent VIN. This information can aid a pathologist in making an accurate diagnosis and may even potentially be of use for the assessment of surgical margins. Studies have shown that patients with HPV-independent VIN in the resection margin have a poorer prognosis [23,24].

Evaluating the CK17 staining patterns, it was observed that in most cases, there was an absence of staining in the basal layers. These CK17-negative basal cell layers often had scant cytoplasm and a small nucleus, consistent with ‘basal-like’ histomorphology. The significance of this pattern is unclear, but one hypothesis is that full-epithelial CK17 expression is increasingly seen in epithelia with greater invasive potential as CK17 can promote propagation and inhibit apoptosis of cells [25]. In squamous cell carcinoma of the anus, esophagus, and oral cavity, CK17 is thought to be associated with disease progression [26,27,28]. Changes in the expression of CK17 that may occur during disease progression may be relevant to establish a prognosis. However, in the current study, a correlation between full thickness CK17 staining and invasive potential could not be demonstrated. The low sample size and influence of subsequent treatment of HPV-independent VIN may be of relevance here.

Despite having used the same scoring methodology as Dasgupta et al. [9,10] in our study, the sensitivity of SOX2 for the diagnosis of HPV-independent VIN was found to be much lower. Also of note is the fact that CK17 and SOX2 were positive in only 14% and 2% of the HSIL cases, respectively, much lower than has previously been reported [20]. Several potential explanations exist for this disparity. Firstly, different clones were used for immunohistochemical staining. Secondly, in our series, no cases adjacent to carcinoma were selected, while this proportion was 52% in the study of Dasgupta.

CK17 and SOX2 displayed a greater specificity for HPV-independent VIN when used in conjunction with p53. Some mimickers of p53 wild-type HPV-independent VIN, such as LS, also frequently showed positive CK17 staining, making CK17 less discriminative between these cases. Positive CK17 in LS was observed in 43% of the cases in this study, compared to in 60 to 90% in the previous reports [11,12,13,14]. The relatively high positivity rate for CK17 in LS is especially problematic when part of the LS epithelium displaying positive CK17 is suspicious for HPV-independent VIN. In our series, all seven LS cases were SOX2 negative, and thus SOX2 could potentially differentiate between CK17-positive LS and CK17-positive HPV-independent VIN. Cook et al. also found no positive SOX2 expression (‘score 3+’) in four LS cases [11]. However, in contrast to these findings, one other study found SOX2 to be negative in only 44% (4/9) of the LS cases [29]. It should be noted that the aforementioned studies, including ours, had a low number of LS cases, making it difficult to draw reliable conclusions. Although our results did not show a high sensitivity for SOX2 as an individual marker, its specificity was high. SOX2, as part of a panel with other stains, may be of additional value in the differential diagnosis between p53 wild-type HPV-independent VIN and (atypical) LS.

The main strength of this study is that it includes a large series of 150 vulvar lesions which were independently assessed by six pathologists. All cases were without concurrent vulvar cancer and selected from a well-defined population-based cohort, reducing the risk of selection bias. The large variety of vulvar lesions in this series, including both HPV-associated and HPV-independent lesions as well as inconclusive cases, provided us with the opportunity to comprehensively assess the markers as initially evaluated by Dasgupta et al. [10]. All 150 cases were stained with the five immunohistochemical markers and agreement was achieved for all diagnoses and interpretations of immunohistochemical stains through multiple consensus meetings.

Our study also has several limitations. Due to the retrospective study design, no clinical data were available when reviewing cases, a limitation that may be particularly important for the optimal interpretation of difficult cases. In addition, only a small subset of the non-dysplastic lesions were cases of LS, while this is amongst the most important differential diagnoses. Additional series including non-dysplastic mimickers of HPV-independent VIN are needed to further explore the diagnostic accuracy of the markers tested here. Although we used the same quantitative cut-offs as Dasgupta et al., a uniform and standardized scoring system for CK17 and SOX2 is not yet available [10]. On the other hand, immunohistochemistry is an ideal technique to evaluate biomarker expression because it is fast, easy, and relatively cheap.

## 5. Conclusions

This study assessed the values of CK17 and SOX2 immunohistochemistry as adjunct diagnostic markers for the accurate diagnosis of HPV-independent VIN. When used in combination with p16 and p53, CK17, in particular, can aid a correct diagnosis. SOX2 can be particularly helpful when there is a differential diagnosis between HPV-independent VIN and LS, but larger studies with more LS cases are needed to confirm this.

## Figures and Tables

**Table 1:** Immunohistochemical expression of p53, CK17, SOX2, p16^INK4a^, and Ki-67 in relation to various categories of vulvar lesions.

Imunohistochemical Marker	Expression Pattern	HPV-Independent VIN				HPV-Associated SIL				Non-Dysplastic		Inconclusive	
		p53 Mutant		p53 Wild-Type		HSIL		LSIL					
		n = 30	(%)	n = 16	(%)	n = 58	(%)	n = 4	(%)	n = 37	(%)	n = 5	(%)
													
**P53**	**Wild-type**	0	(0)	16	(100)	58	(100)	4	(100)	37	(100)	4	(80)
	**Mutant**	30	(100)	0	(0)	0	(0)	0	(0)	0	(0)	1	(20)
													
**CK17**	**Negative**	8	(27)	0	(0)	50	(86)	4	(100)	27	(73)	2	(40)
	No expression	0	(0)	0	(0)	16	(28)	2	(50)	17	(46)	1	(20)
	Patchy	8	(27)	0	(0)	34	(59)	2	(50)	10	(27)	1	(20)
	**Positive**	22	(73)	16	(100)	8	(14)	0	(0)	9	(24)	3	(60)
	Full-epithelial	8	(27)	6	(38)	1	(2)	0	(0)	3	(8)	1	(20)
	Partial thickness	14	(47)	10	(63)	7	(12)	0	(0)	6	(16)	2	(40)
	**Not judgeable**	0	(0)	0	(0)	0	(0)	0	(0)	1	(3)	0	(0)
													
**SOX2**	**Negative**	17		14		57		4	(100)	35	(95)	5	(100)
	No expression	11	(37)	9	(56)	53	(91)	3	(75)	24	(65)	2	(40)
	Patchy	6	(20)	5	(31)	4	(7)	1	(25)	11	(30)	3	(60)
	**Positive**	13	(43)	2	(13)	1	(2)	0	(0)	1	(3)	0	(0)
	Full-epithelial	8	(27)	0	(0)	1	(2)	0	(0)	0	(0)	0	(0)
	Partial thickness	5	(17)	2	(13)	0	(0)	0	(0)	1	(3)	0	(0)
	**Not judgeable**	0	(0)	0	(0)	0	(0)	0	(0)	1	(3)	0	(0)
													
**P16^INK4a^**	**Negative**	29	(97)	16	(100)	0	(0)	3	(75)	36	(97)	5	(100)
	**Block-positive**	1	(3)	0	(0)	58	(100)	1	(25)	0	(0)	0	(0)
	**Not judgeable**	0	(0)	0	(0)	0	(0)	0	(0)	1	(3)	0	(0)
													
**Ki-67**	**Not increased**	4	(13)	2	(13)	0	(0)	0	(0)	20	(54)	1	(20)
	**Increased**	26	(87)	14	(88)	58	(100)	4	(100)	17	(46)	4	(80)

**Table 2:** Test characteristics of p53, CK17, and SOX2 immunohistochemistry in HPV-independent VIN. Non-dysplastic lesions with a valid immunohistochemical result were used as control group. NB: not corrected for disease prevalence.

HPV-Independent VIN	Immunohistochemical Marker	Sensitivity		Specificity		Accuracy	
		% (95% CI)		% (95% CI)		% (95% CI)	
**All**	**P53**	65	(50–79)	100	(90–100)	80	(71–89)
n = 46	**CK17**	83	(69–92)	75	(58–88)	79	(69–87)
	**SOX2**	33	(20–48)	97	(85–99.9)	61	(70–88)
	**P53/CK17**	100	(92–100)	76	(59–88)	89	(80–95)
	**P53/SOX2**	70	(54–82)	95	(82–99)	81	(71–89)
	**P53/CK17/SOX2**	100	(92–100)	70	(53–84)	87	(78–93)
							
**P53 mutant**	**CK17**	73	(54–88)	75	(58–88)	74	(62–84)
n = 30	**SOX2**	43	(26–63)	97	(85–99.9)	73	(60–83)
							
**P53 wild-type**	**CK17**	100	(79–100)	75	(58–88)	83	(70–92)
n = 16	**SOX2**	13	(2–38)	97	(85–99.9)	71	(57–83)

## Data Availability

Data can be made available upon reasonable request.
